# Switch Between El Nino and La Nina is Caused by Subsurface Ocean Waves Likely Driven by Lunar Tidal Forcing

**DOI:** 10.1038/s41598-019-49678-w

**Published:** 2019-09-11

**Authors:** Jialin Lin, Taotao Qian

**Affiliations:** Atmospheric Science Program, The Ohio State University, Columbus, USA

**Keywords:** Atmospheric dynamics, Natural hazards, Physical oceanography

## Abstract

The El Nino-Southern Oscillation (ENSO) is the dominant interannual variability of Earth’s climate system, and strongly modulates global temperature, precipitation, atmospheric circulation, tropical cyclones and other extreme events. However, forecasting ENSO is one of the most difficult problems in climate sciences affecting both interannual climate prediction and decadal prediction of near-term global climate change. The key question is what cause the switch between El Nino and La Nina. For the past 30 years, ENSO forecasts have been limited to short lead times after ENSO sea surface temperature (SST) anomaly has already developed, but unable to predict the switch between El Nino and La Nina. Here, we demonstrate that the switch between El Nino and La Nina is caused by a subsurface ocean wave propagating from western Pacific to central and eastern Pacific and then triggering development of SST anomaly. This is based on analysis of all ENSO events in the past 136 years using multiple long-term observational datasets. The wave’s slow phase speed and decoupling from atmosphere indicate that it is a forced wave. Further analysis of Earth’s angular momentum budget and NASA’s Apollo Landing Mirror Experiment suggests that the subsurface wave is likely driven by lunar tidal gravitational force.

## Introduction

The 1876–1877 eastern hemisphere drought and resultant Great Famine caused a death toll of 17 million people in China, India, Indonesia, Australia and South Africa, and prompted the discovery of ENSO^1–3^. ENSO is a 3–6 year oscillation of Earth’s climate system, which is the first principle component of global monthly sea surface temperature anomaly, and contributes 18% of the total variance^4–8^. ENSO strongly modulates global temperature^9^, precipitation^10^, droughts^11^, tropical cyclones^12^, tornadoes^13^, extratropical cyclones^14^ and other extreme events^15^, and also plays an important role in global warming projections^16–18^.

However, forecasting ENSO is one of the most difficult problems in atmospheric sciences^19–22^. The long-lasting unanswered question is what cause the switch between El Nino and La Nina. For the past 30 years, ENSO forecasts have been limited to short lead time of 6–9 months after ENSO sea surface temperature anomaly has already developed (Supplementary Fig. 1). Most of the ENSO forecast models cannot predict the switch between El Nino and La Nina^19–22^ which requires a lead time of 12 months or longer (Supplementary Fig. 2). This is the case not only for statistical models, but also for most of the dynamical coupled general circulation models (CGCMs). State-of-the-art CGCMs have substantial difficulty in simulating the correct oscillation period and amplitude of ENSO^16,23^, which is connected to their biases in simulating tropical mean state and ocean-atmosphere feedbacks^24,25^. This affects not only their ENSO predictions, but also their decadal to multi-decadal predictions of near-term global climate change^26–30^.

The existing ENSO theories can be categorized into six groups^31–33^ (Supplementary Fig. 3) including (1) slow coupled mode theories^3,34–36^, (2) stochastic forcing theories^37,38^, (3) recharge oscillator theory^39^, (4) delayed oscillator theory^40–42^, (5) advective-reflective oscillator theory^43^, and (6) western Pacific oscillator theory^44^. Ocean-atmosphere feedback mechanisms are emphasized by the first three theories, but coupled climate models with ocean-atmosphere feedbacks still have difficulty in simulating ENSO and are quite sensitive to different physical parameterizations. Free ocean waves, including equatorial Kelvin and Rossby waves, are emphasized by the other three theories, and have been found in both observations^45–49^ and models^41,50–54^. The phase speeds of the free Kelvin waves are generally 2–3 m/s, while those of the free Rossby waves are 0.5–1 m/s. These waves are driven by anomalous westerly or easterly winds^55^, which are often associated with the intraseasonal Madden-Julian Oscillation (MJO)^56–59^, and show clear horizontal and vertical propagations associated with different types of El Nino^60^. However, the propagation speeds of free ocean waves are too fast to explain the 3–6 year time-scale of ENSO.

Sea surface state variables, including sea surface temperature (SST), sea level pressure (SLP), surface winds and sea surface height (SSH), are the predictors generally used by statistical ENSO models, and also serve as initial fields for dynamical ENSO models. However, during the transition phase between El Nino and La Nina, which is often called “neutral phase”, the SST, SLP, SSH and surface wind anomalies are very weak, and cannot provide good predictors for long-lead ENSO prediction.

Here, we demonstrate that the switch between El Nino and La Nina is caused by a subsurface ocean wave propagating from western Pacific to central/eastern Pacific, and then trigger the development of sea surface temperature anomaly there. This is based on analysis of all ENSO events in the past 136 years using multiple long-term observational datasets. See Methods section for detailed information about the datasets and methods.

## The Subsurface Ocean Wave Associated with ENSO Lifecycle

Supplementary Fig. 4 shows vertical cross-section of climatological mean ocean subsurface temperature along the equator averaged between 5N-5S for three observational datasets: (a) TAO buoy array for 23 years (1993–2015), (b) UKMO ocean analysis for 61 years (1955–2015), and (c) SODA ocean reanalysis for 133 years (1880–2012). All datasets show the well-defined temperature contrast between western Pacific warm pool and eastern Pacific cold tongue. The white line is the climatological 23.5 °C line, which is a good representation of thermocline. Supplementary Fig. 5 shows the climatological mean vertical velocity along the equator. Because the equatorial upwelling is driven by the trade winds, there is strong upwelling to the east of dateline from the thermocline to 10 m. The upwelling to the west of dateline is much weaker.

**Figure 1:**
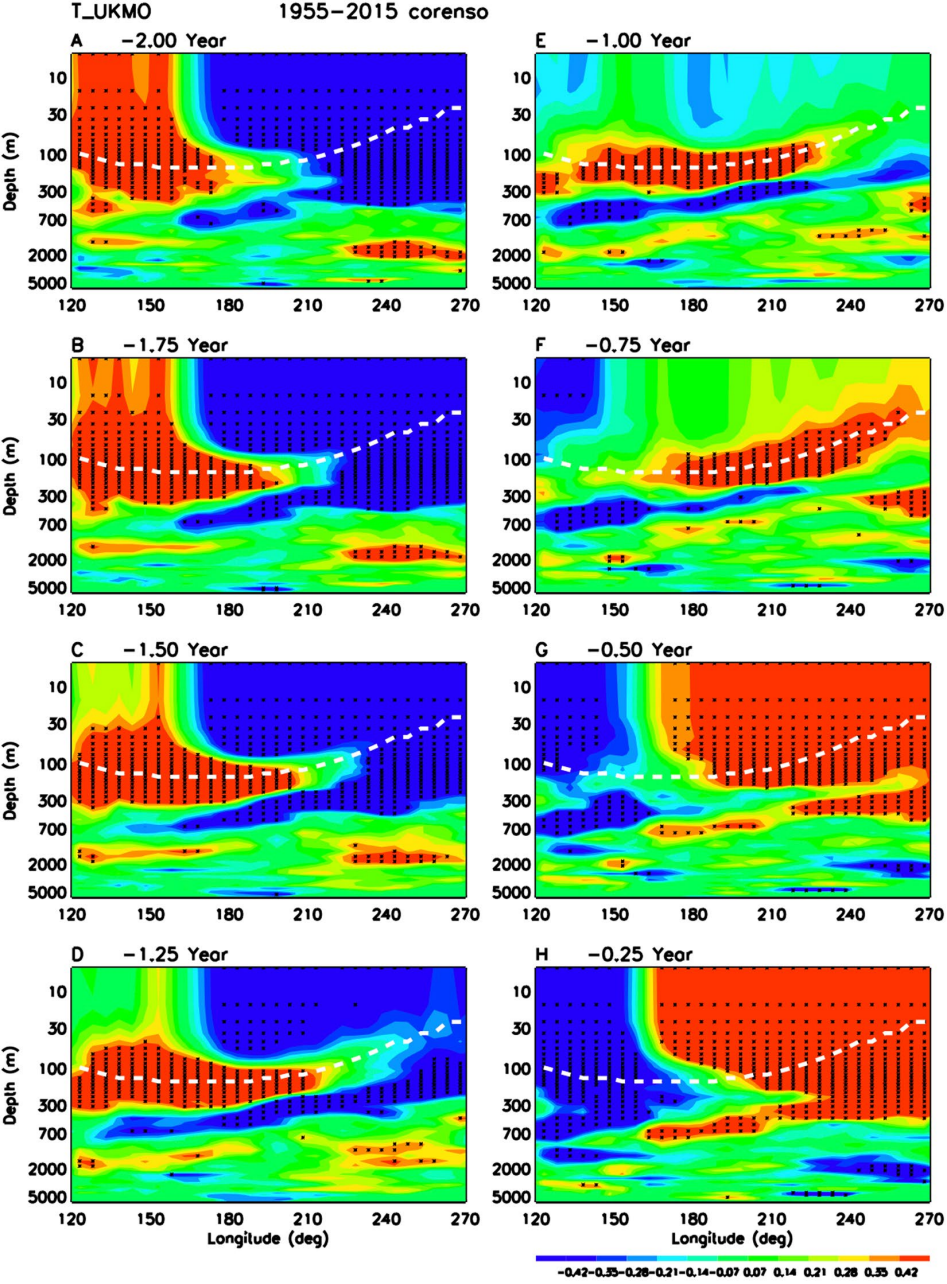
Eastward propagation of ocean subsurface wave leading to switch from La Nina to El Nino. Shadings show lag-correlation of UKMO ocean analysis subsurface temperature along the equator (5N-5S) with Nino3.4 SST from (**A**) −24 months to (**H**) −3 months for all ENSO events in 61 years from 1955–2015. Black stars denote the grids with lag-correlation above 95% confidence level. The white dashed line is the climatological 23.5 °C line from Supplementary Fig. 4b.

Figure 1 illustrates the lag-correlation of UKMO ocean analysis subsurface temperature with Nino3.4 SST from (a) −24 months (La Nina) to (h) −3 months (3 months before El Nino) for all ENSO events in 61 years from 1955–2015. Figure 1 demonstrates three key points. First, there is a clear subsurface ocean wave propagating eastward along the thermocline from western Pacific to central and eastern Pacific (Fig. 1A–F). The warm temperature anomaly already starts off from western Pacific at the peak of La Nina (Fig. 1A), and quickly passes the dateline within 3 months when the surface temperature in central and eastern Pacific still shows significant cold anomaly of La Nina (Fig. 1B). Secondly, as soon as the ocean wave passes the dateline and enters the eastern Pacific (Fig. 1B), the strong mean-state upwelling in eastern Pacific starts to advect the warm temperature anomaly from the thermocline (at 30–120 meters depth in the eastern Pacific) towards the surface, with a vertical speed of 2–10 meters per month (Supplementary Fig. 5). The warm advection starts earlier in central Pacific (Fig. 1B), but the thermocline is deeper there and it takes more time for the warm anomaly to reach the surface. The warm advection starts much later in far east Pacific close to the coast of South America (Fig. 1F), but the thermocline is much shallower there and the warm anomaly can quickly reach the surface. This warm advection likely contributes to the decay of La Nina from −21 months to −12 months (Fig. 1B–E), and then initiates the warm SST anomalies and triggers the Bjerknes feedback, leading to development of El Nino at −6 months to −3 months (Fig. 1G,H). The corresponding amplitude of temperature variations is above 1 °C (not shown), which is similar to the amplitude in the delayed oscillator model^41^, and thus sufficient to cause the switch. Thirdly, during the neutral transition phase at −12 months and −9 months (Fig. 1E,F), there is no significant surface temperature anomaly, but the subsurface ocean wave anomaly is highly significant and provides an excellent predictor for ENSO forecast.

**Figure 2:**
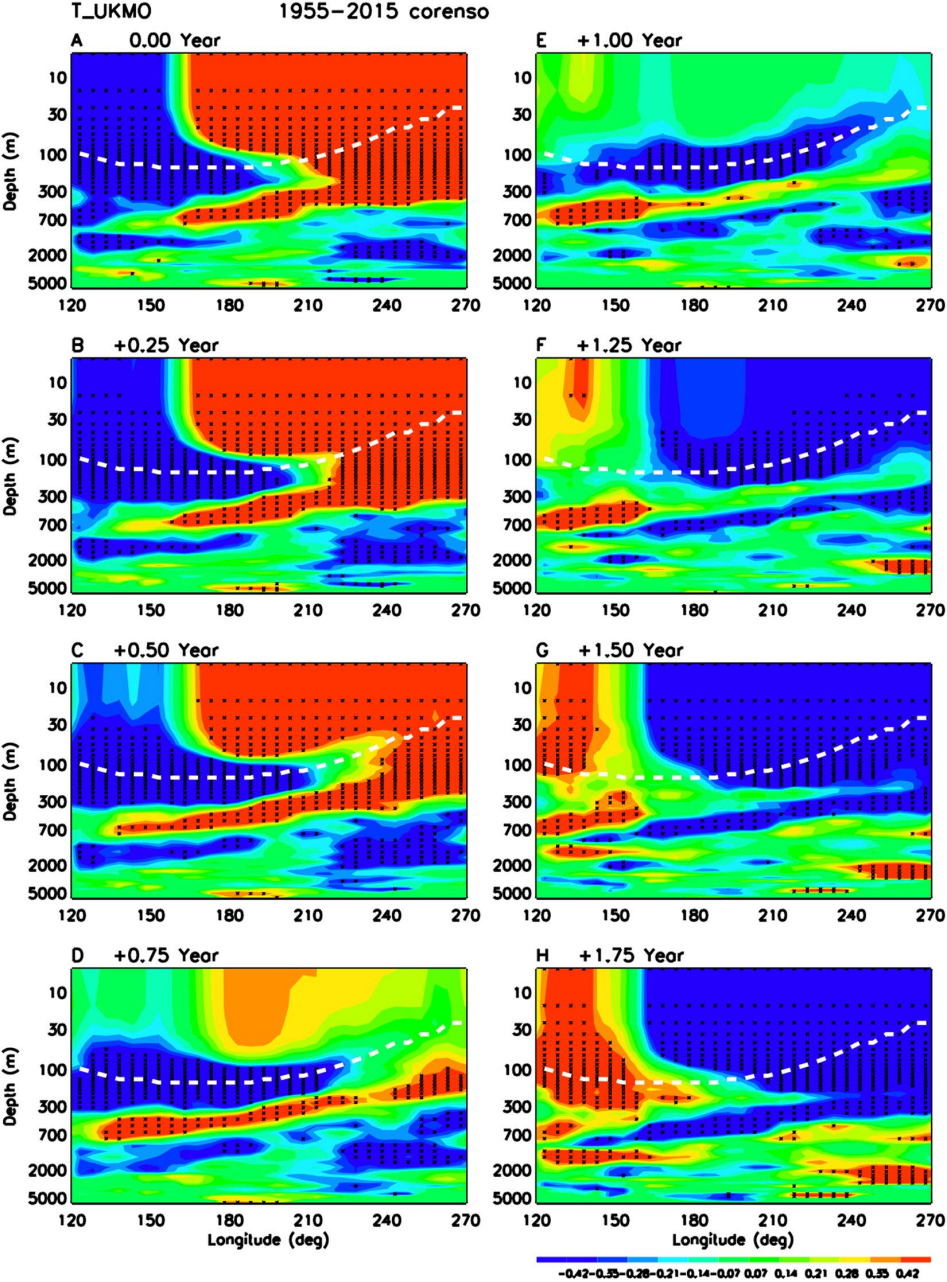
Same as Fig. 1 but for switch from El Nino to La Nina for (**A**) 0 month (El Nino) to (**H**) +21 months after El Nino.

The switch from El Nino to La Nina is shown in Fig. 2. Again, at the peak of El Nino when the entire central and eastern Pacific are occupied by significant warm surface temperature anomalies (Fig. 2A), cold subsurface ocean wave has already started off from western Pacific. When the surface temperature anomalies have disappeared during the neutral transition phase from +9 months (Fig. 2D) to +15 months (Fig. 2F), cold subsurface ocean wave anomaly is highly significant, providing important predictors for the forthcoming La Nina.

Similar results are obtained from 33 years (1993–2015) of raw TAO buoy data (Supplementary Figs 6 and 7). Extending our analysis to all ENSO events in 133 years (1880–2012) using SODA ocean reanalysis, which assimilates all available ocean subsurface temperature observations, also reveals the same results (Supplementary Figs 8 and 9). Therefore, the eastward propagation of subsurface ocean wave associated with ENSO lifecycle is a highly robust physical phenomena.

## What Drives the Subsurface Ocean Wave?

**Figure 3:**
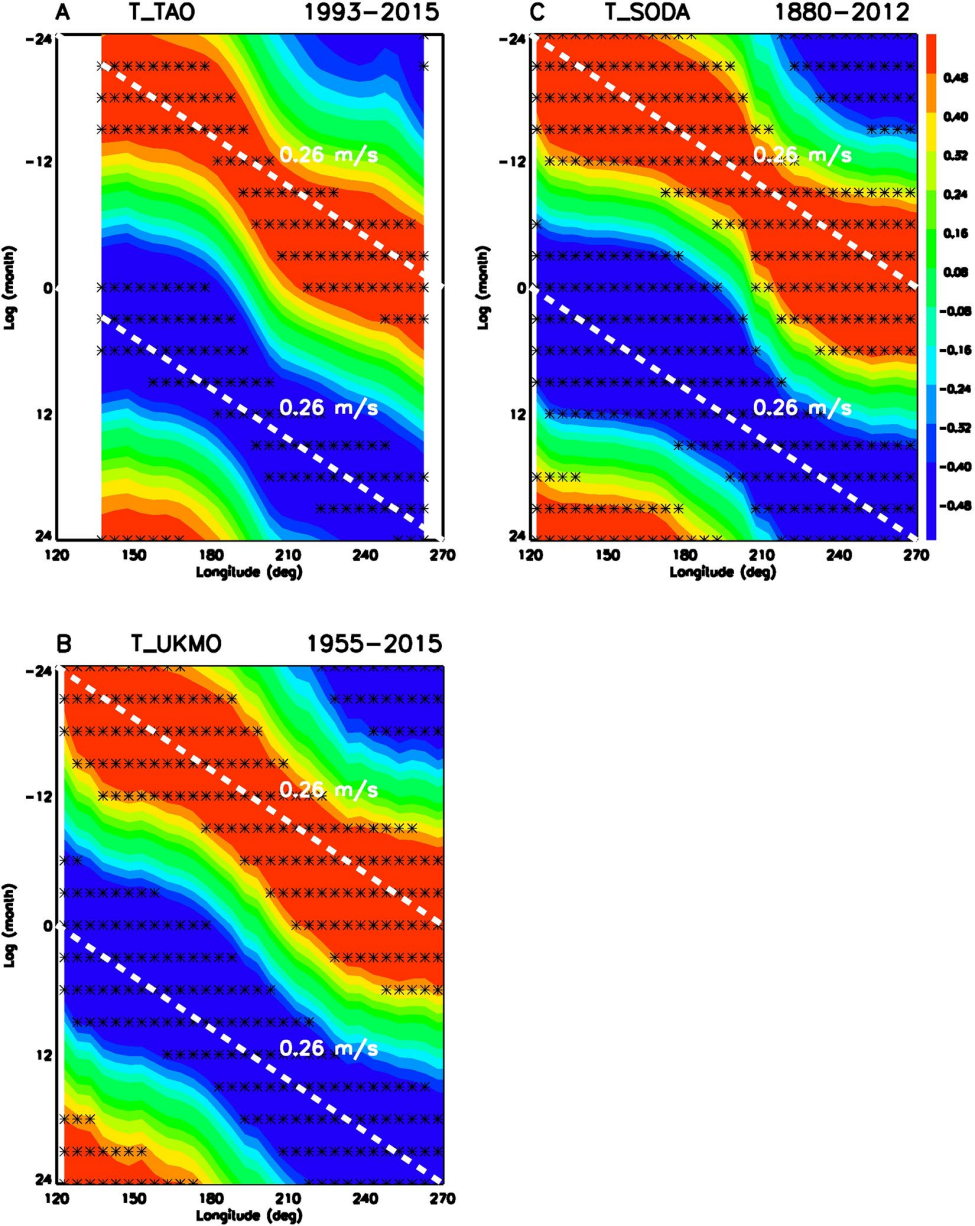
Eastward propagation of ocean subsurface wave along the thermocline associated with ENSO lifecycle in three observational datasets. (**A**) TAO buoy array for 23 years (1993–2015), (**B**) UKMO ocean analysis for 61 years (1955–2015), and (**C**) SODA ocean reanalysis for 133 years (1880–2012). Shadings show lag-correlation with Nino3.4 SST for ocean temperature averaged between 5N-5S along the thermocline (climatological 23.5 °C depth). Black stars denote the grids with lag-correlation above 95% confidence level. White dashed lines are the 0.26 m/s phase speed line.

There are three types of ocean waves: free ocean wave, free ocean-atmosphere coupled wave, and forced ocean wave. First, we determine if the observed wave is a free ocean wave by calculating the phase speed of propagation. Figure 3 provides a summary of wave propagation along the thermocline for all three observational datasets. All three datasets consistently demonstrate an eastward propagation with a phase speed of 0.2–0.3 m/s, which is much slower than the phase speeds of free ocean waves^40^. The free Kelvin waves driven by westerly wind bursts associated with the MJO generally have a phase speed of 2–3 m/s^56–59^, which is an order of magnitude larger than the phase speed of the wave found here. Therefore, the observed wave is not a free ocean wave.

The subsurface ocean wave is not likely a free ocean-atmosphere coupled slow mode because during the neutral transition phases (Figs 1E–F and 2D–F), there is no significant SST anomaly and the strong subsurface ocean wave is totally decoupled from the atmosphere. During these periods, the wave still keeps the slow phase speed of 0.2–0.3 m/s (Fig. 3) and thus is not a free wave emanating from the source region.

The third possibility is forced ocean wave. The major external forcing for ocean is the tidal gravitational force. The thermocline is associated with the strongest vertical temperature gradient and thus tend to show the largest temperature anomaly when driven by tidal vertical motion, which is consistent with the depth of the subsurface ocean wave. The moon’s revolution around the Earth is from the west to the east in the same direction as the Earth’s rotation, which is consistent with the eastward propagation of the subsurface ocean wave. Connection between the observed subsurface wave with tide is also supported by the evolution of zonal mean ocean temperature associated with ENSO lifecycle (Supplementary Fig. 10), which can be compared with subsurface wave propagation along the equator (Fig. 1). When the warm subsurface wave propagates from western Pacific to central Pacific (Fig. 1A–D), zonal mean temperature shows clear warm anomaly at the 100–300 m depth of the wave (Supplementary Fig. 10a–d). When the subsurface wave rises up in eastern Pacific and triggers the El Nino (Fig. 1E–H), zonal mean temperature shows clear process that the wave breaks through the cold temperature anomaly of La Nina, and pushes it away from the equator in both hemispheres (Supplementary Fig. 10e–h). SODA ocean reanalysis shows similar results for all ENSO events in 133 years (Supplementary Fig. 11). The zonal mean structure is similar to the lunar semidiurnal tides in the ocean^61^ and atmosphere^62^. Because the tidal force at the equator doubles that at the pole, the largest amplitude occurs in the tropics. For a vertically propagating gravity wave, upward (downward) phase propagation implies downward (upward) energy dispersion. For the lunar atmospheric tide, the forcing is strongest at the Earth’s surface where rising sea level forces the atmosphere, so tidal energy disperses upward and phase propagates downward^62^. In contrast, for the lunar oceanic tide, the forcing is strongest at the ocean surface because tidal force increases with the distance to the center of the Earth and vertical displacement is largest at the top of tidal bulge^63^, so tidal energy disperses downward and phase propagates upward.

Is there lunar tidal forcing at ENSO’s time-scale? The three commonly used ENSO indices consistently demonstrate that the generally-thought wide spectral peak of ENSO between 3–7 years in fact consists of two main spectral peaks at 3 years and 6 years, respectively (Supplementary Fig. 12). Lunar tidal gravitational force calculated from NASA Apollo Landing Mirror Experiment^64,65^ and Earth’s angular momentum budget consistently show two sharp peaks at 6 years and 9 years, respectively (Supplementary Fig. 13). The western Pacific subsurface temperature at the thermocline depth also demonstrates sharp 6-year and 9-year peaks (Supplementary Fig. 14), suggesting a strong link between the lunar tidal force and the Earth’s ocean subsurface temperature. The 6-year peak of lunar tidal force matches very well with the 6-year component of ENSO. Lag-correlations between the 6-year component of lunar tidal forcing with equatorial ocean subsurface temperature demonstrate clearly the subsurface ocean wave propagating from western Pacific to central and eastern Pacific and triggering SST anomaly there, suggesting that the 6-year component of lunar tidal forcing drives the 6-year component of ENSO (Supplementary Figs 15 and 16).

The 6-year and 9-year peaks of lunar tidal forcing are key lunar tidal constituents at the interannual time-scale^65–67^, although the 6-year component did not draw much attention in research. The three different lunar months: draconic (nodal passage: 27.212208 days), sidereal (inertial space period: 27.321661 days), and anomalistic (perigee to perigee: 27.554551 days) combine to give periods of 6.00 years, 8.85 years and 18.6 years^65–67^. Global mean surface temperature demonstrates 6-year and 9-year oscillations, which have been proposed to be driven by lunar tidal forcing^67,68^. The 3-year component of ENSO may be generated by the sub-harmonics of the 6-year tidal forcing, or the interactions of 6-year and 9-year forcings with seasonal cycle and other high-frequency oscillations.

The observed oscillation periods of ENSO are irregular, which are known to be affected by the background state associated with longer-period oscillations and ocean-atmosphere feedback^23^. The tidal forcing in real world is also “irregular” because it is contributed by many tidal constituents. For example, in order to predict the day-to-day sea level variations along the coast, at least 10 dominant tidal constituents in the diurnal, semidiurnal and quarter-diurnal bands are needed in the global tidal models^69^. Another example is that the interannual lunar tidal forcing is dominated by the 6-year component between the 1920s and 1940s, but dominated by the 9-year component during other time periods, which coincide well with similar oscillations in observed global mean surface temperature^67^. Therefore, tidal forcing may also contribute to the observed irregularity of ENSO. In addition, after the El Nino has developed (Figs 1G,H and 2A), equatorial upwelling driven by tidal forcing may affect the amplitude of El Nino.

**Figure 4:**
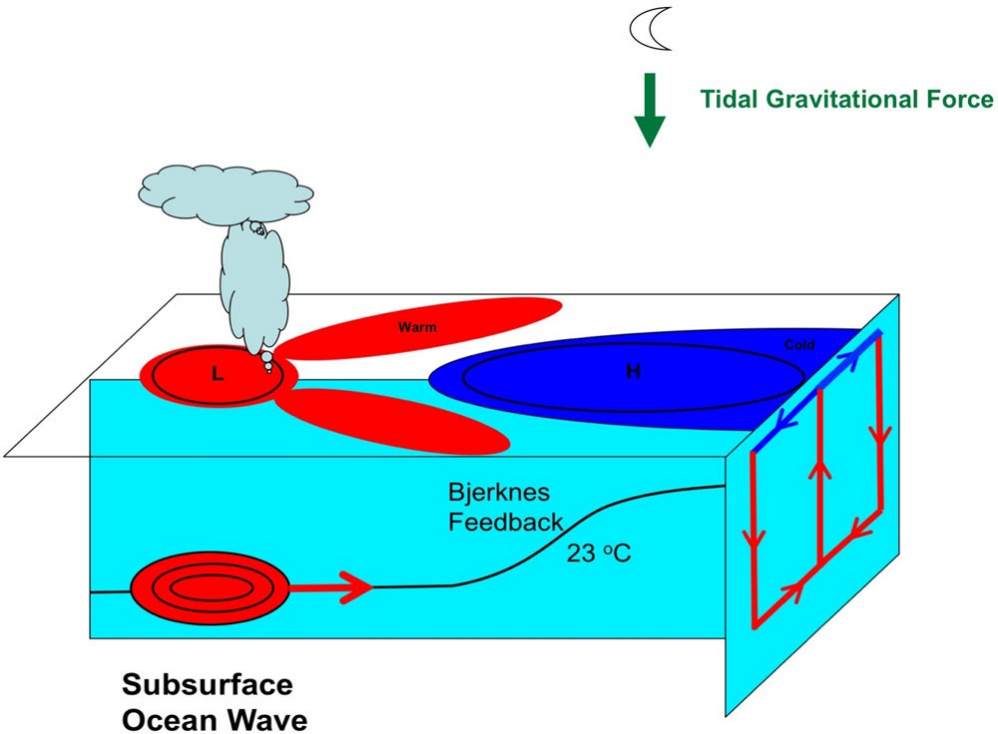
Schematic depiction of the physical mechanisms leading to the switch between El Nino and La Nina.

Our key findings are summarized schematically in Fig. 4. We have demonstrated highly robust evidence that the switch between El Nino and La Nina is caused by an ocean subsurface wave propagating along thermocline from western Pacific to central and eastern Pacific, and then triggering the development of SST anomaly there. Our findings suggest two possible ways to improve the current ENSO forecasts: (1) Adding the subsurface ocean wave to statistical ENSO forecast models and improving its representation in CGCMs, which may lead to an improvement of the 12-month ENSO forecast. Right now, none of the statistical models considers the subsurface ocean wave. In fact, the only two ENSO forecast models that can make good 12-month forecast, the NASA GMAO model and GFDL FLOR model (Supplementary Fig. 2), are assimilating carefully subsurface temperature. (2) Adding lunar tidal forcing to statistical models and CGCMs may provide important long-range predictability. Currently, the ocean-atmosphere coupled runs of climate models, such as the IPCC models historical runs and projection runs^23,70^, are called “free runs” and are not expected to capture the timing of ENSO events in the real world. Adding lunar tidal forcing may help to simulate the correct timing of ENSO events, in addition to improving the simulated oscillation period and amplitude of ENSO. Recently, the ocean modelling community show strong interest in lunar tidal forcing because there are more and more evidences that tidal mixing plays a key role in global ocean circulation^71–73^. Parameterizations of diurnal and semidiurnal tidal mixing have been implemented into several OGCMs such as the GFDL MOM^74^, HYCOM^75^, and MIROC^76^. However, for simulating the interannual tidal components related to ENSO, explicit modelling of time-varying gravitational field is needed. An exciting new progress is that the MPI OM group has developed a tidal forcing option to include explicit time-varying gravitational forcing from the Sun and Moon including the seasonal, annual, interannual and inter-decadal tidal cycles^77^. For each time step of simulation, the actual positions of the Sun and Moon are calculated using the semi-analytic planetary theory Variations Seculaires des Orbites Planetaires (VSOP87)^78^, and the associated gravitational forcing is determined. This tidal forcing option has not been used in the MPI model’s climate predictions^79^ or IPCC runs^80^. Nevertheless, the MPI model has demonstrated that it is possible to add to GCMs explicit time-varying gravitational forcing from the Sun and Moon. The VSOP87 source code is available online (http://neoprogrammics.com/vsop87/), and we hope that the climate modelling community could install it to the climate models and conduct long-term coupled ocean-atmosphere experiments, which may provide insights on the relationship between tidal forcing and ENSO as suggested by the our observational study. If the model experiments confirm that lunar tidal forcing drives the observed subsurface ocean waves leading to the switch between El Nino and La Nina, this new physics will provide valuable long-range predictability, and help to improve the ENSO forecasts and decadal to multi-decadal predictions of global climate change^26–30^.

## Methods

Datasets used in this study are listed in Table 1. The main ENSO index used in this study is Nino3.4 SST from ERSST dataset. Linear trend and composite seasonal cycle are first removed from all datasets. Maximum entropy spectrum is calculated following Press and Flannery^81^. The anomalies are then filtered with a 3–6 year butterworth filter (Murakami)^82^. Lag-correlation is calculated with the ENSO index. Statistical significance is evaluated following Oort and Yienger^83^.

**Table 1:** Datasets used in this study.

Variables	Datasets	Time period	Reference
Current ENSO Forecasts by All Models	International Research Institute (IRI) ENSO Forecast Archive	1992–2018	https://iri.columbia.edu/forecast/ensofcst/Data/archive/
**Sea Surface Temperature**	Extended Reconstructed Sea Surface Temperature (ERSST) Version 4	1880–2016	Huang *et al*.^86^
Ocean Subsurface Temperature	Tropical Atmosphere Ocean (TAO) buoy	1993–2015	McPhaden^87^
	United Kingdom Meteorological Office (UKMO) EN4 Analysis	1955–2015	Good *et al*.^88^
	Simple Ocean Data Assimilation (SODA) Version 2.2.4	1880–2012	Giese *et al*.^89^
Sea Level Pressure, Upper Air Temperature, Geopotential Height, Winds	European Centre for Medium-Range Weather Forecasts (ECMWF) ERA-Interim Reanalysis	1979–2016	Dee *et al*.^90^
	National Center for Environmental Prediction (NCEP) Reanalysis	1948–2016	Kalnay *et al*.^91^
	National Oceanic and Atmospheric Administration (NOAA) 20th Century Reanalysis (20Cv2)	1880–2012	Compo *et al*.^92^
Moon-Earth Distance	NASA Apollo Landing Mirror Experiment/Lunar Laser Ranging Measurements	1970–2017	http://polac.obspm.fr/llrdatae.html
Earth’s Rotation and Length of The Day	International Earth Rotation and Reference Systems (IERS)	1623–2016	https://www.iers.org

The anomalies are also filtered with a 6-year butterworth filter and lag-correlation is calculated with the lunar tidal gravitational force. Lunar tidal gravitational force is calculated from two sources. The first is direct calculation from Moon-Earth distance measured by NASA’s Apollo Landing Mirror experiment from 5 mirrors on the Moon deployed by Apollo 11 and others. The second is by calculating the angular momentum of whole Earth system, which is anti-correlated with lunar tidal friction. We calculated the whole atmospheric angular momentum using 6-hourly NCEP reanalysis upper air winds for all levels around the globe for 69 years (1948–2016) following Weickmann and Berry^84^, and solid Earth angular momentum from Earth’s rotation speed (length of the day measurement) following Rosen *et al*.^85^.

## Supplementary information

Supplementary Information

## Data Availability

Datasets used in this study are from NOAA PMEL TAO Buoy Website, NOAA ESRL climate data archive and NCAR Research Data Archive.
