# Supplementary material for: Switch Between El Nino and La Nina is Caused by Subsurface Ocean Waves Likely Driven by Lunar Tidal Forcing
Source: Sci Rep. 2019 Sep 11;9:13106. doi: 10.1038/s41598-019-49678-w (PMC6739352; doi:10.1038/s41598-019-49678-w)
Supplement: Supplementary file 1 — Supplementary Information [file 41598_2019_49678_MOESM1_ESM.pdf]

Switch Between El Nino and La Nina is Caused by Subsurface Ocean Waves Likely Driven by Lunar Tidal Forcing: Supplementary Information

Jialin Lin[1,*] and Taotao Qian[1]

[1]*Atmospheric Science Program, The Ohio State University, U.S.A.*
**Corresponding author (email: lin.789@osu.edu)*

Contents of this file

Supplementary Figures 1-16

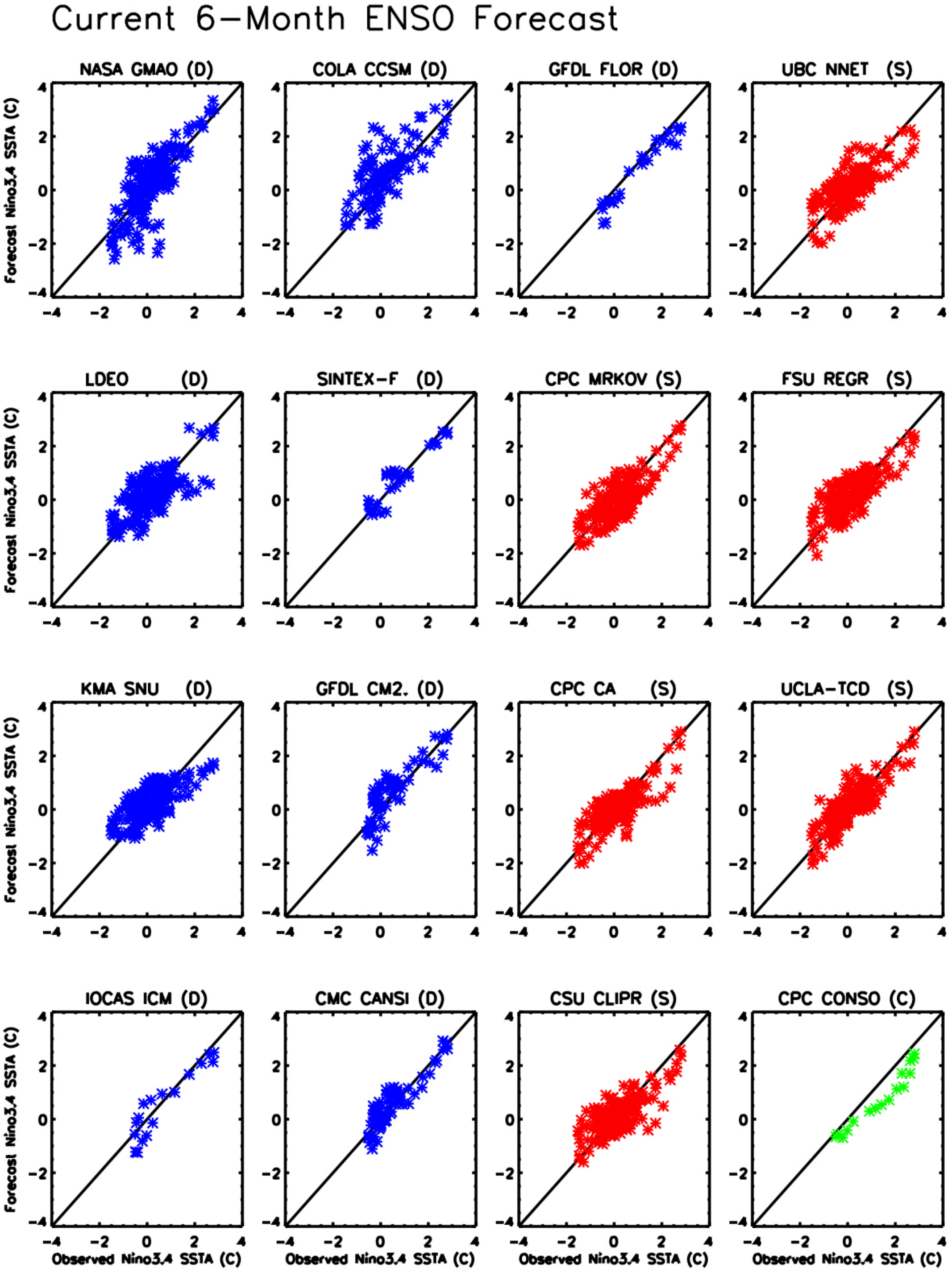

Supplementary Figure 1. 6-month ENSO forecast from February 2002 to August 2018 by all ENSO forecast models, including statistical models (red), dynamical models (blue) and CPC CONSOL (green).

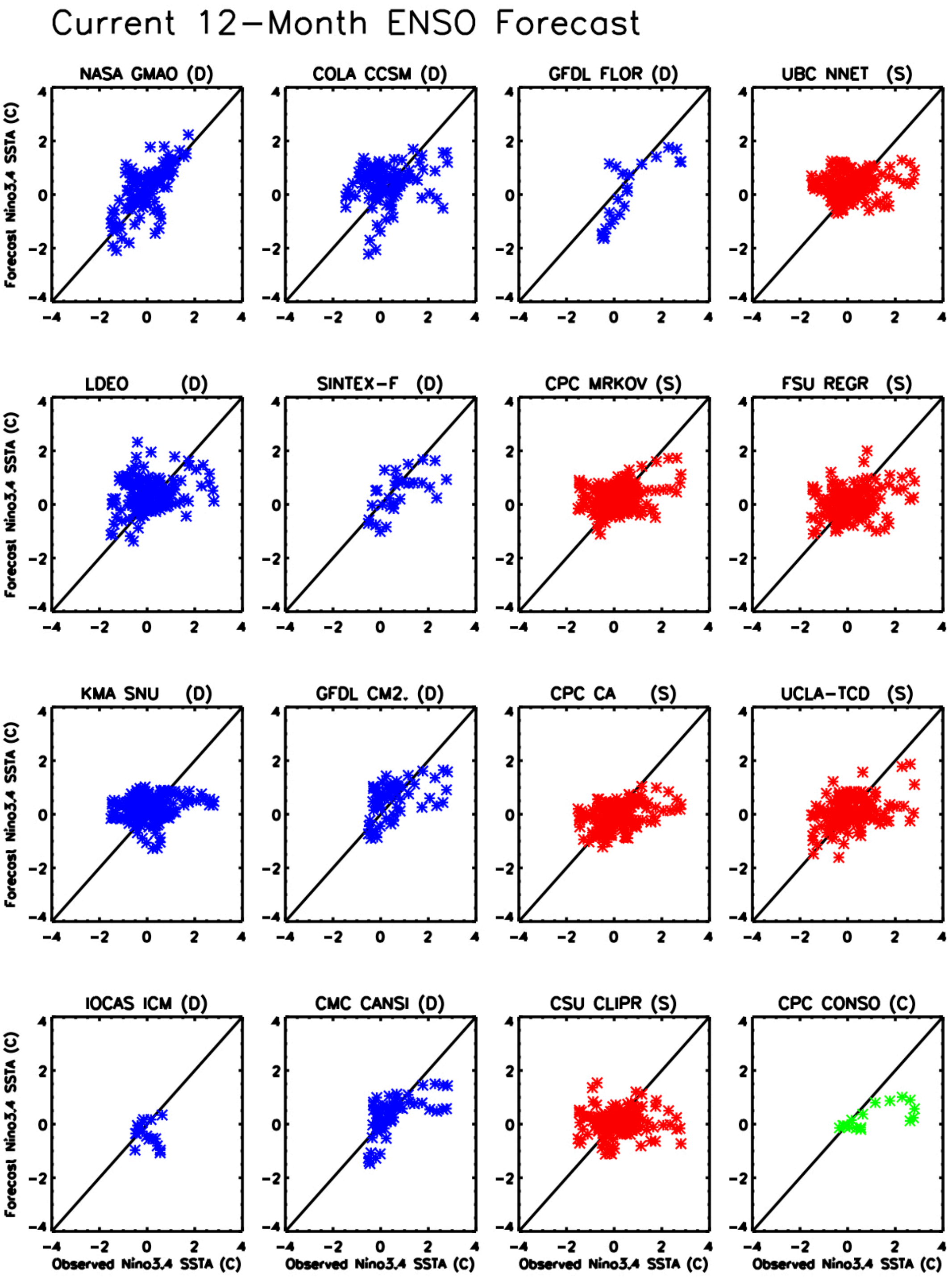

Supplementary Figure 2. Same as Supplementary Figure 1 but for current 12-month ENSO forecast.

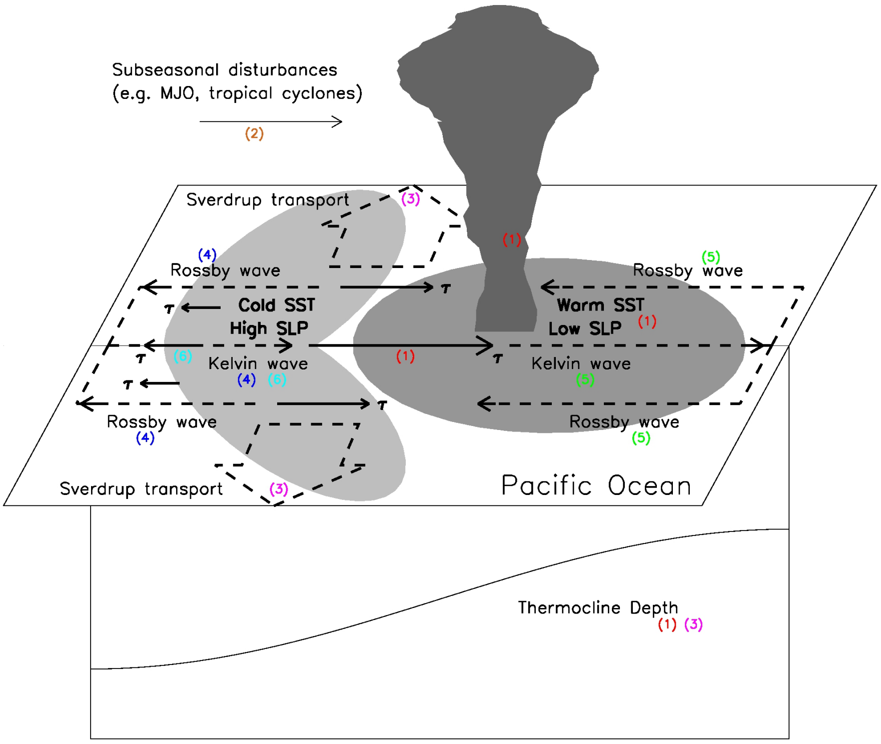

Supplementary Figure 3. Schematic of existing ENSO theories. The numbers in color denote that the corresponding physical process is emphasized by one or more of the existing theories, including (1) slow coupled mode theory, (2) stochastic forcing theory, (3) recharge oscillator theory, (4) delayed oscillator theory, (5) advective-reflective oscillator theory, and (6) western Pacific oscillator theory. See text for details (modified from Lin 2009, reference 34).

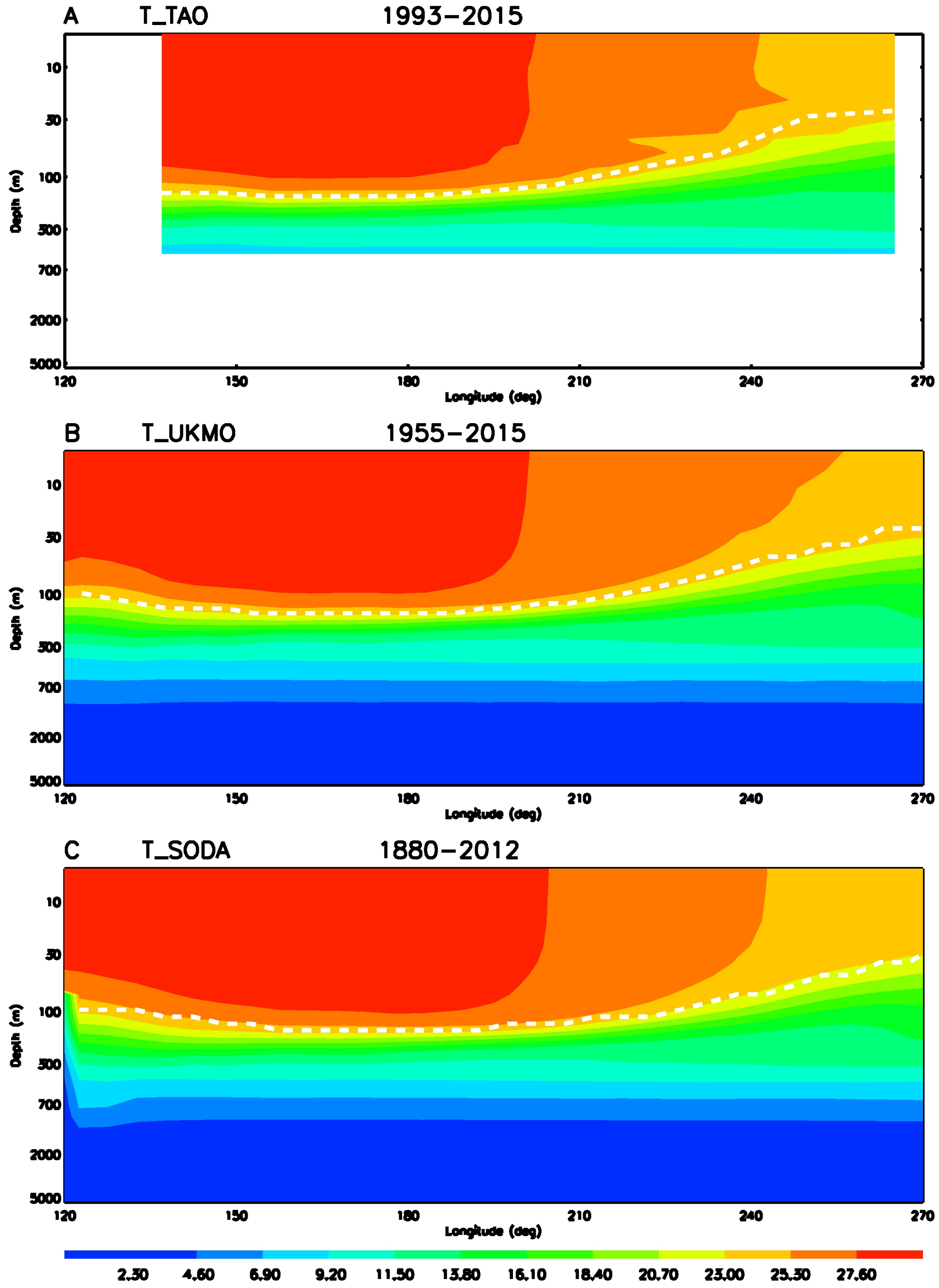

Supplementary Figure 4. Vertical cross-section of climatological mean ocean subsurface temperature along the equator averaged between 5N-5S. Shadings show the temperature. (A) TAO buoy array (1993-2015). (B) UKMO ocean analysis (1955-2015). (C) SODA ocean reanalysis (1880-2012). The white dashed line is the climatological 23.5 °C line.

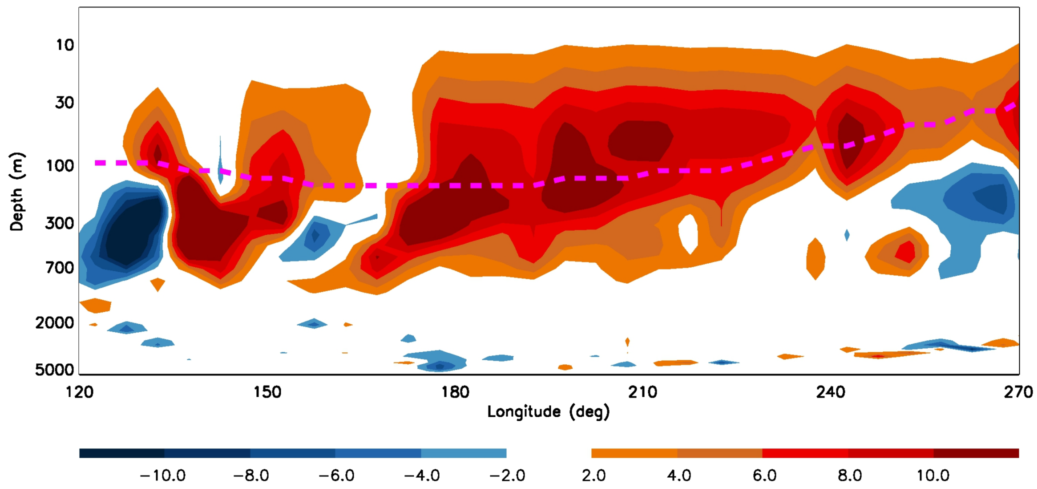

Supplementary Figure 5. Vertical cross-section of climatological mean ocean vertical velocity along the equator averaged between 5N-5S from SODA ocean reanalysis (1880-2012). The unit is m/month. The magenta dashed line is the climatological 23.5 °C line.

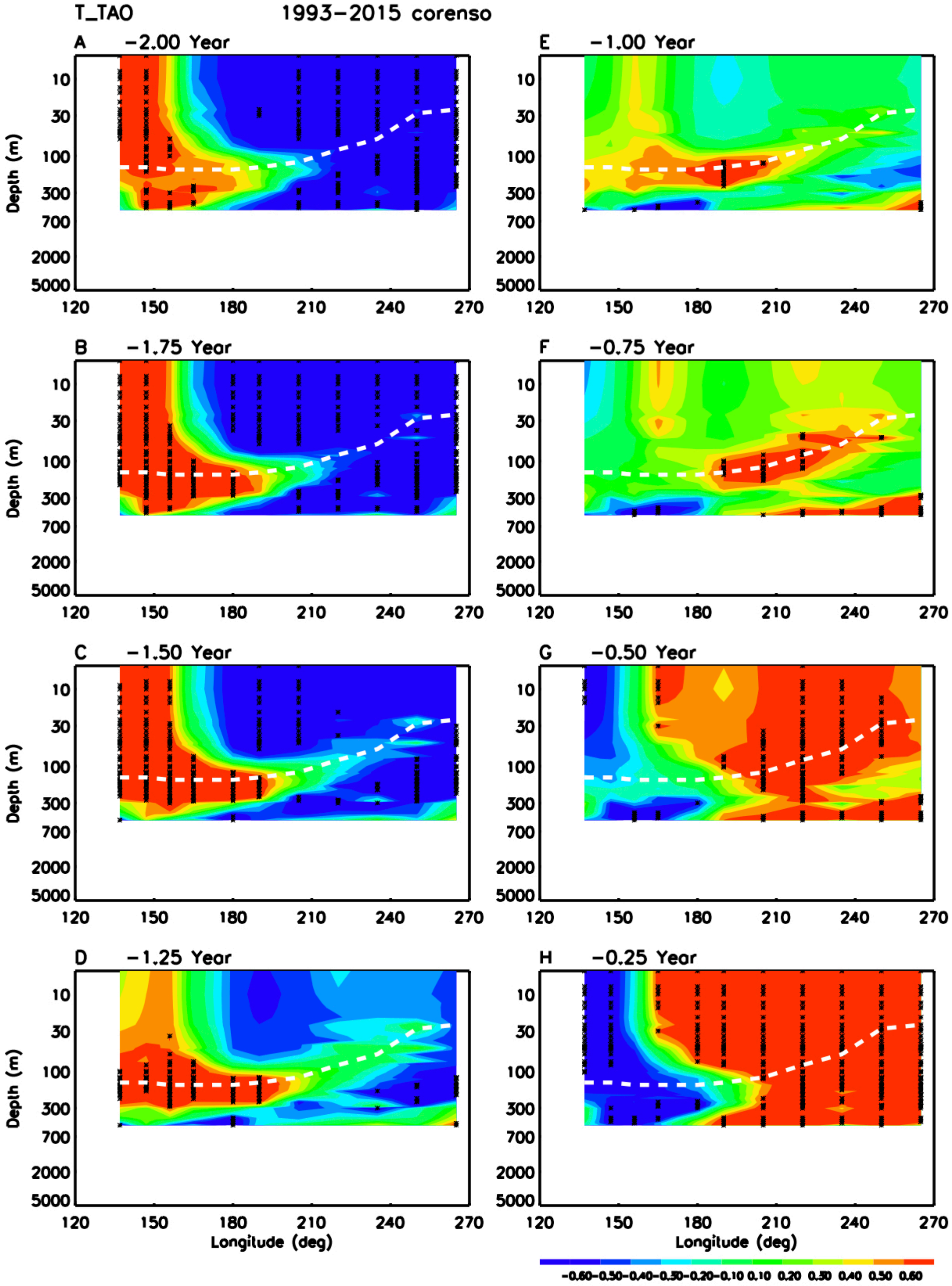

Supplementary Figure 6. Same as Figure 1 but for TAO buoy array data for 1993-2015.

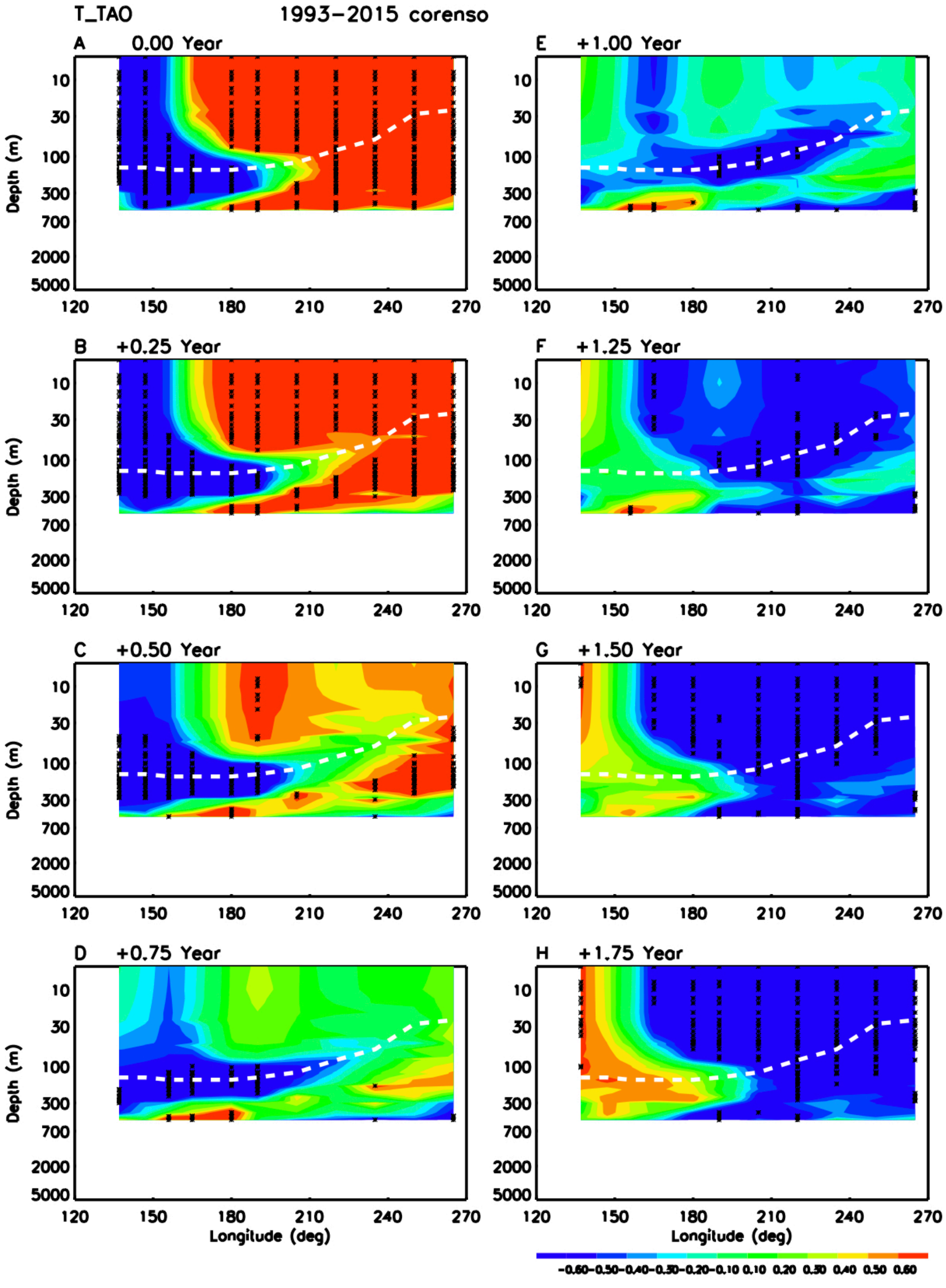

Supplementary Figure 7. Same as Figure 2 but for TAO buoy array data for 1993-2015.

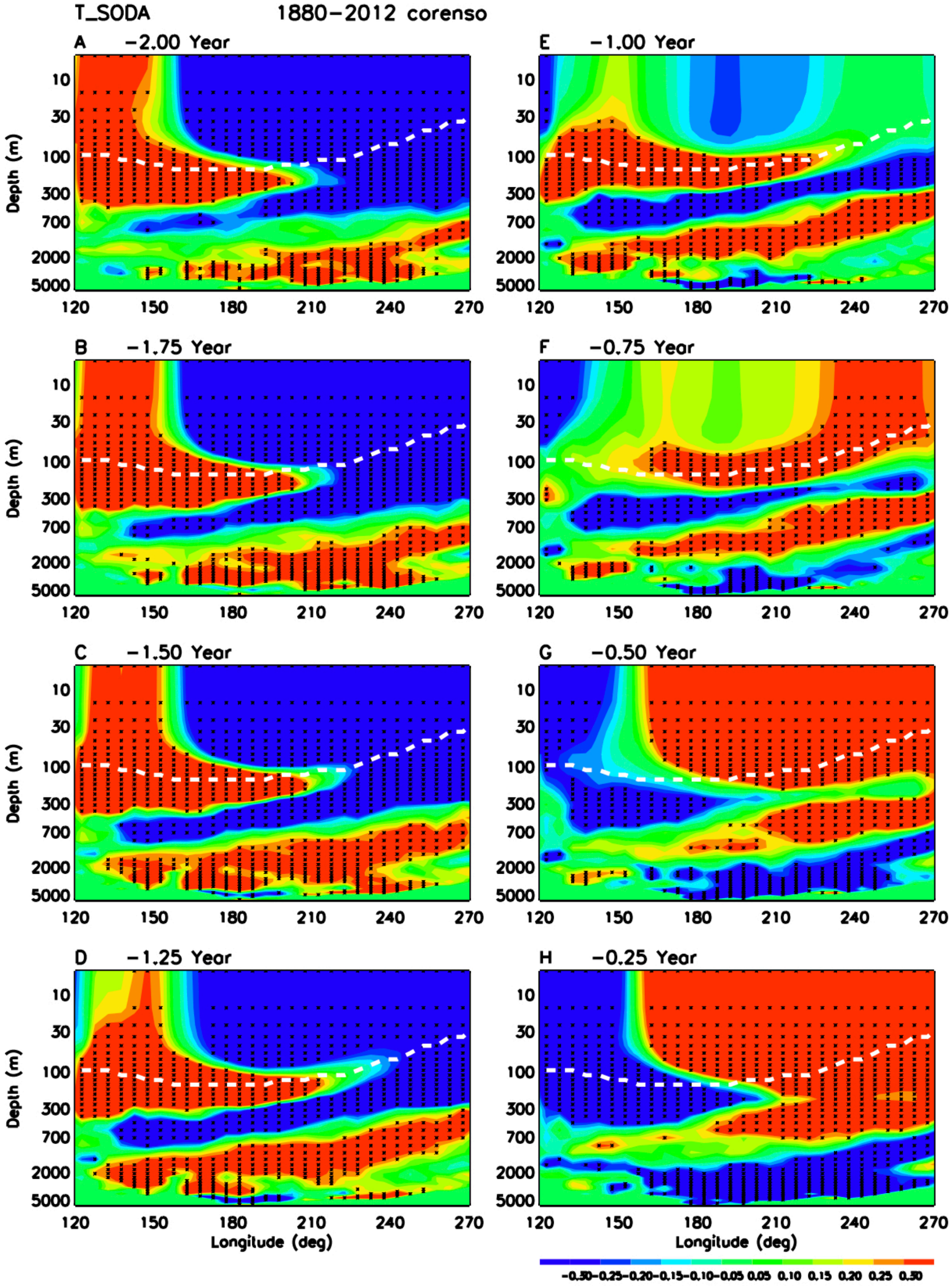

Supplementary Figure 8. Same as Figure 1 but for SODA ocean reanalysis for 1880-2012.

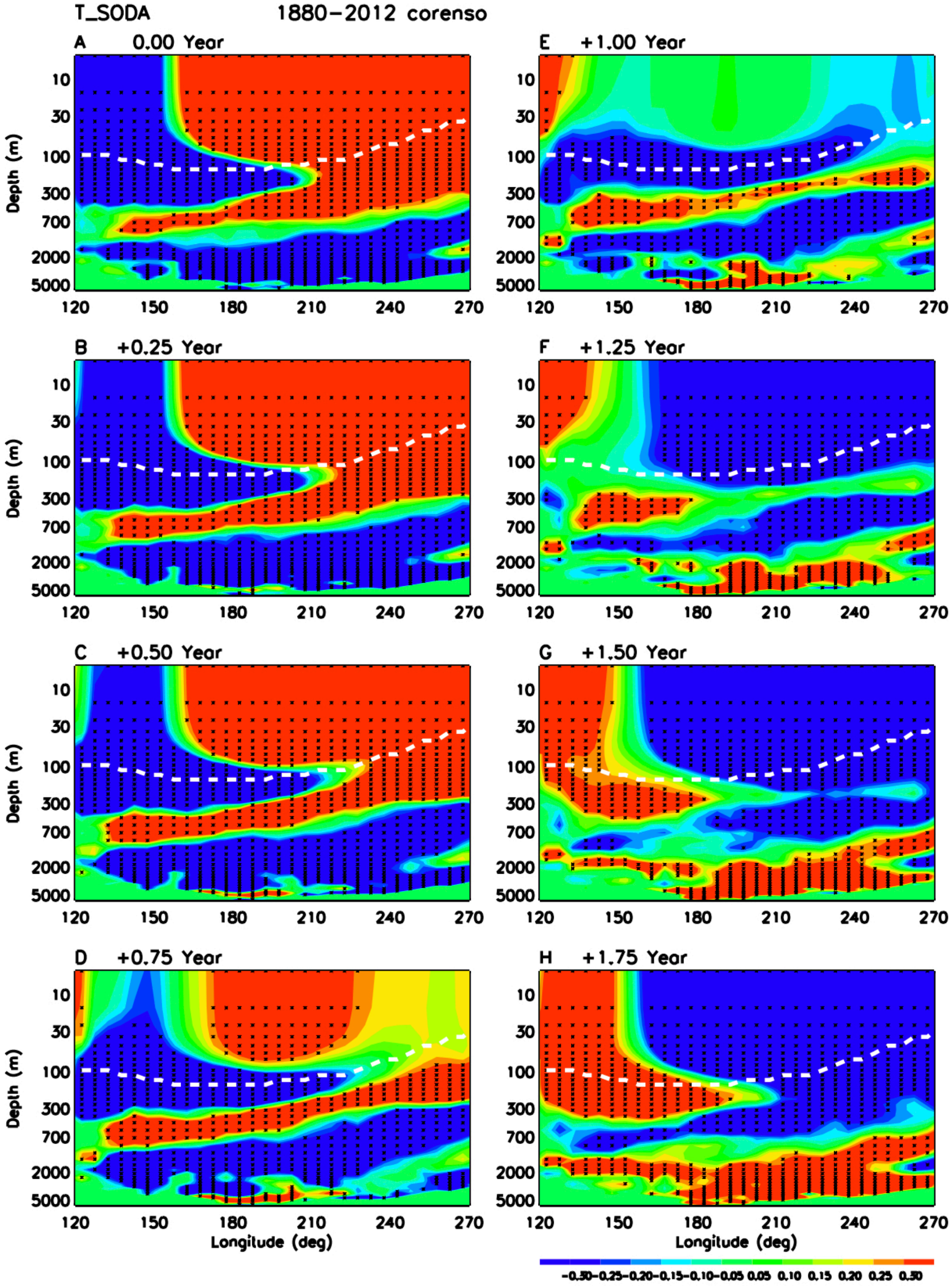

Supplementary Figure 9. Same as Figure 2 but for SODA ocean reanalysis for 1880-2012.

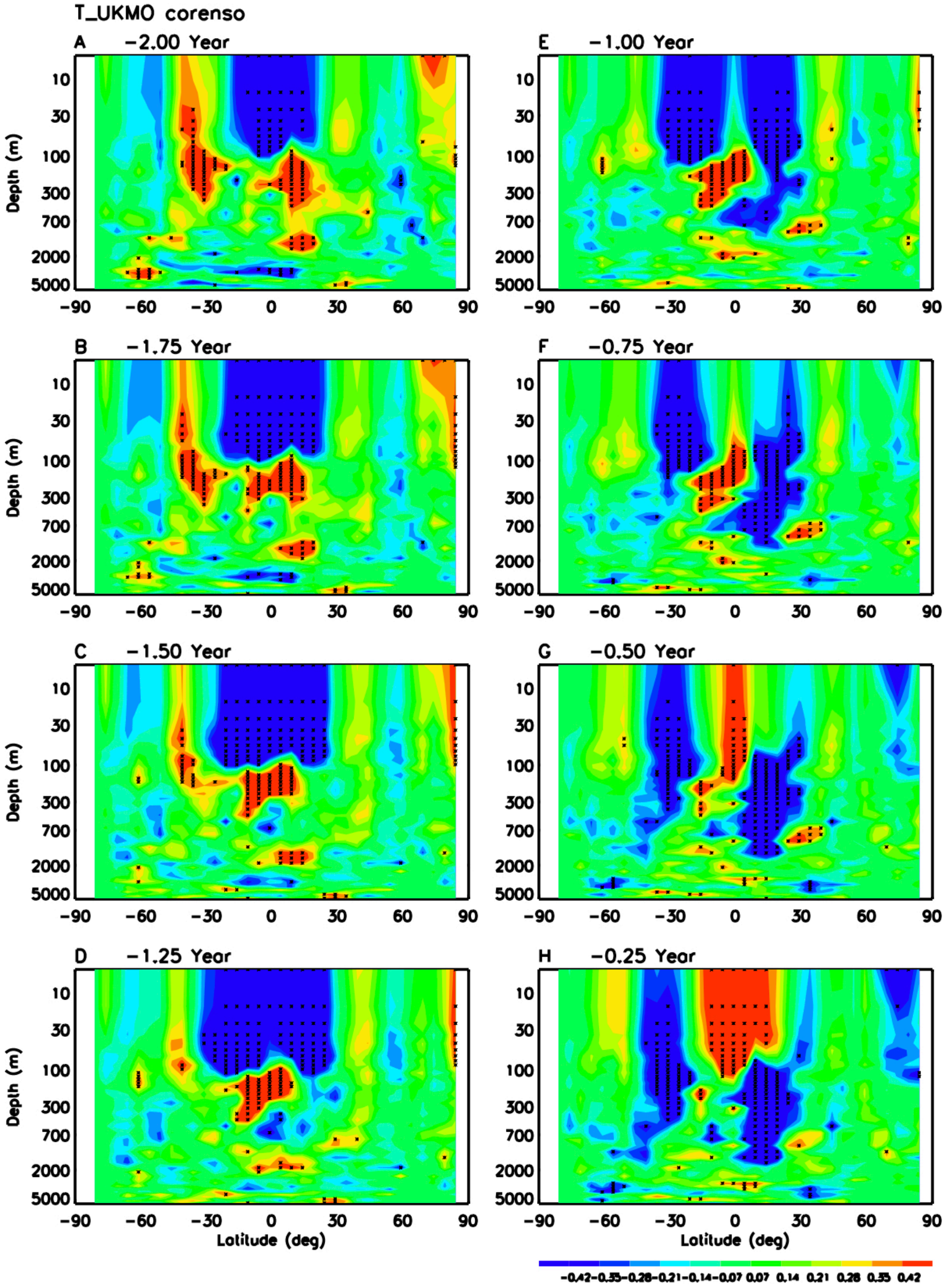

Supplementary Figure 10. Evolution of zonal mean ocean temperature with ENSO lifecycle. Shadings show lag-correlation of UKMO ocean analysis zonal mean temperature with Nino3.4 SST from (A) -24 months to (H) -3 months for all ENSO events in 61 years (1955-2015). Black stars denote the grids with lag-correlation above 95% confidence level.

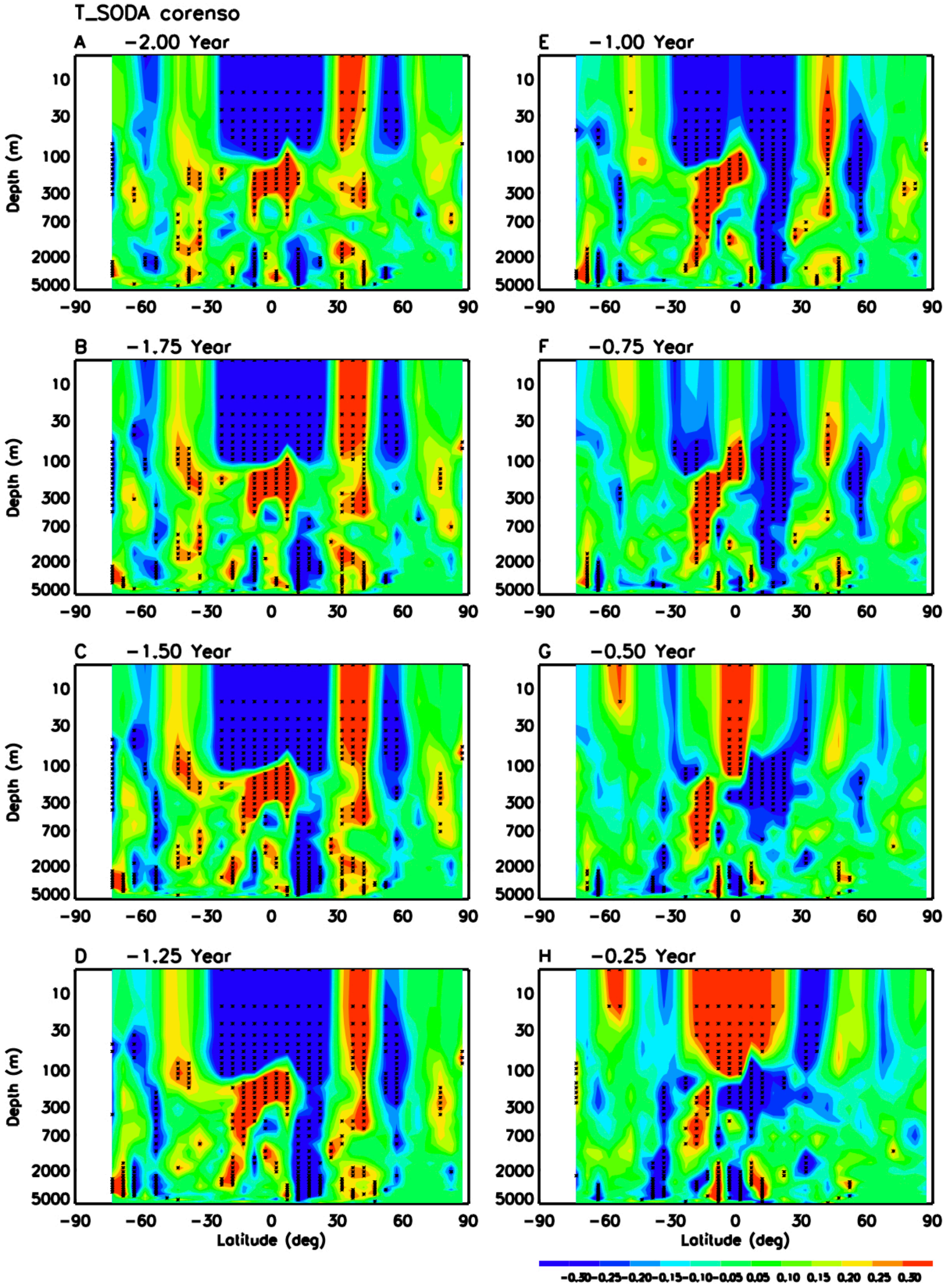

Supplementary Figure 11. Same as Supplementary Figure 10 but for SODA ocean reanalysis for all ENSO events in 133 years (1880-2012).

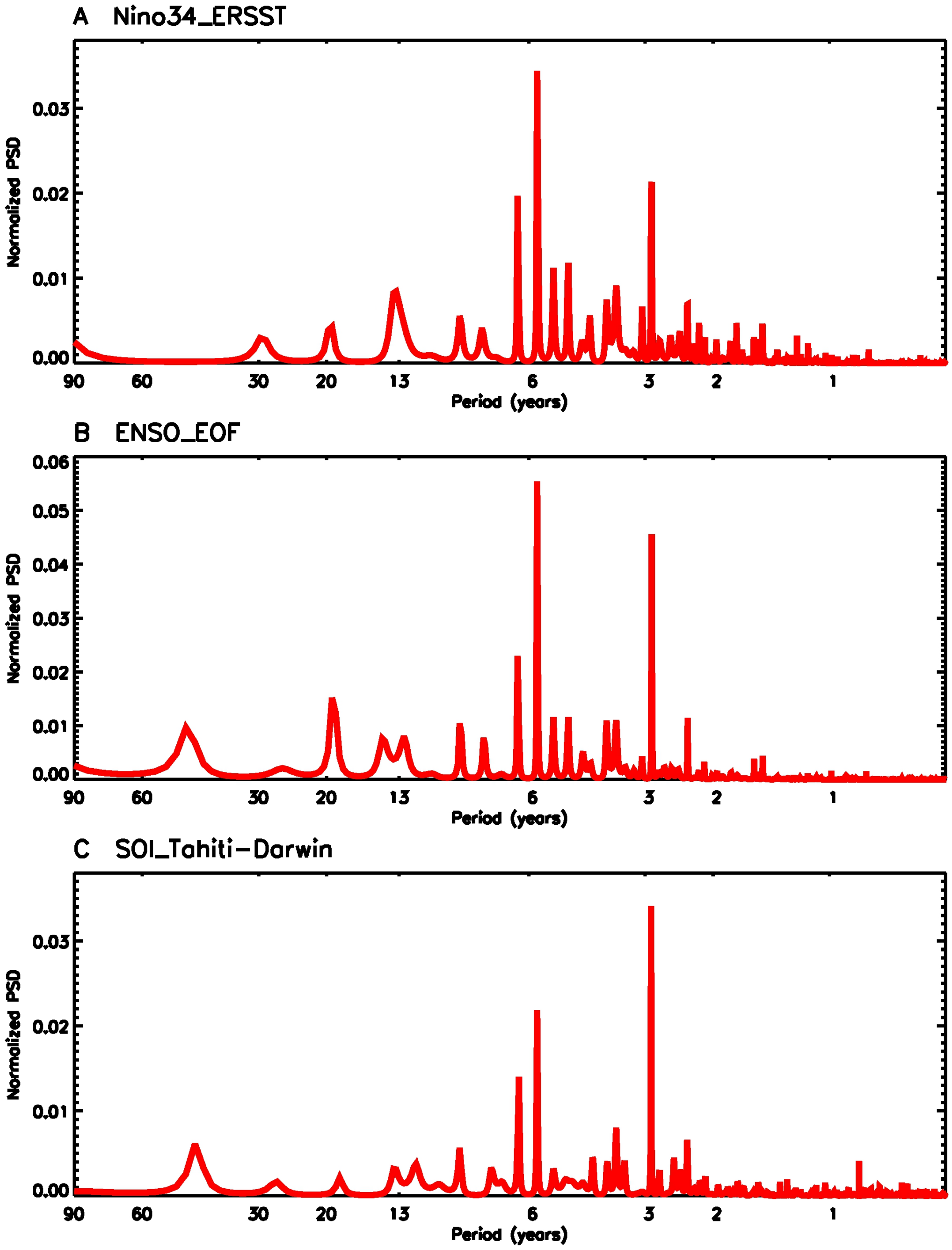

Supplementary Figure 12. Maximum entropy spectrum of three commonly used ENSO indices. (A) Nino3.4 SST, (B) EOF1 of global monthly SST anomaly, and (C) Southern Oscillation Index (SLP difference between Tahiti and Darwin).

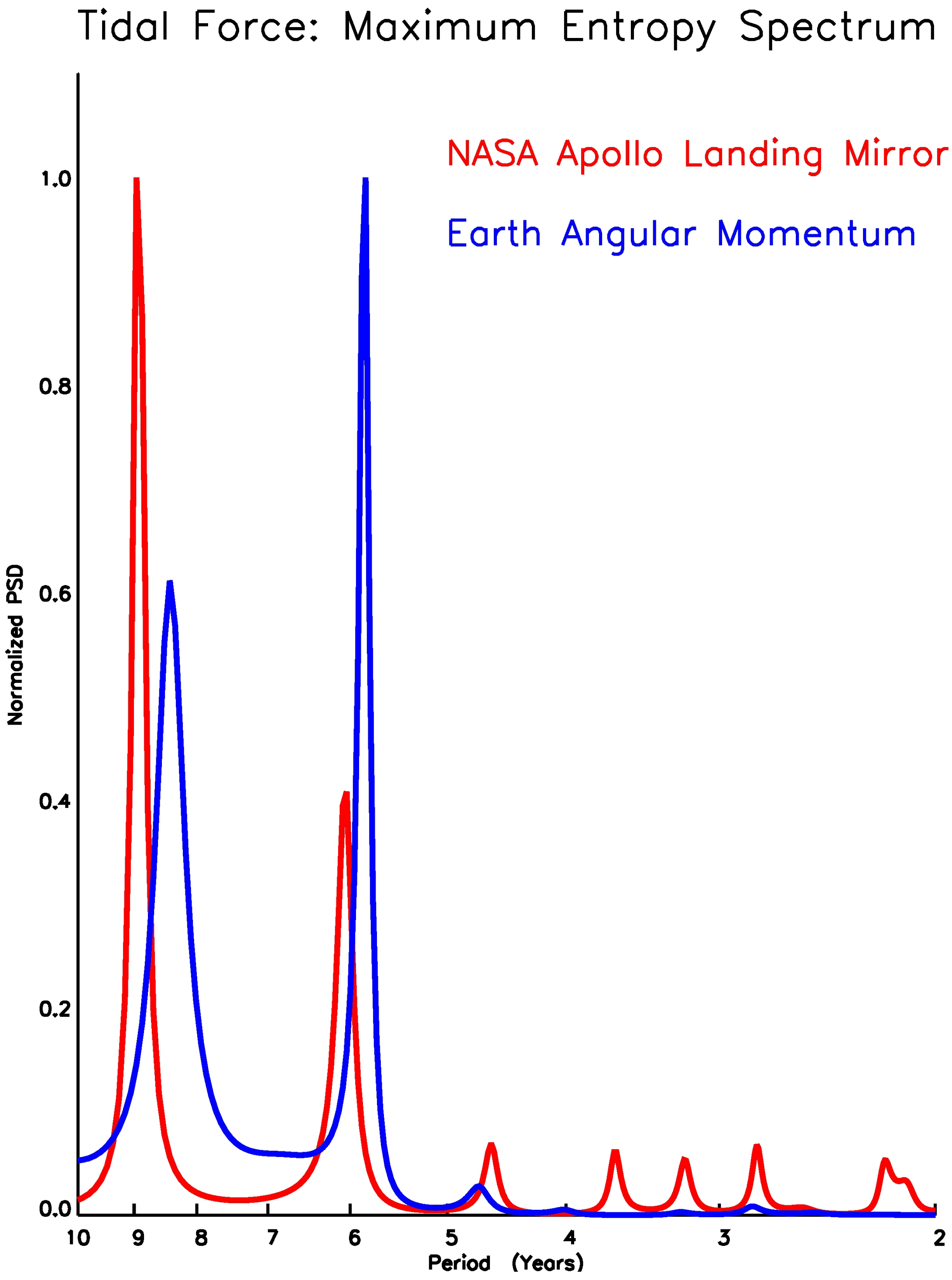

Supplementary Figure 13. Maximum entropy spectrum of lunar tidal gravitational forcing from two data sources: Moon-Earth distance measurements from NASA's Apollo Landing Mirror Experiment (red) and Earth's angular momentum calculated from NCEP reanalysis and Earth's rotation (blue).

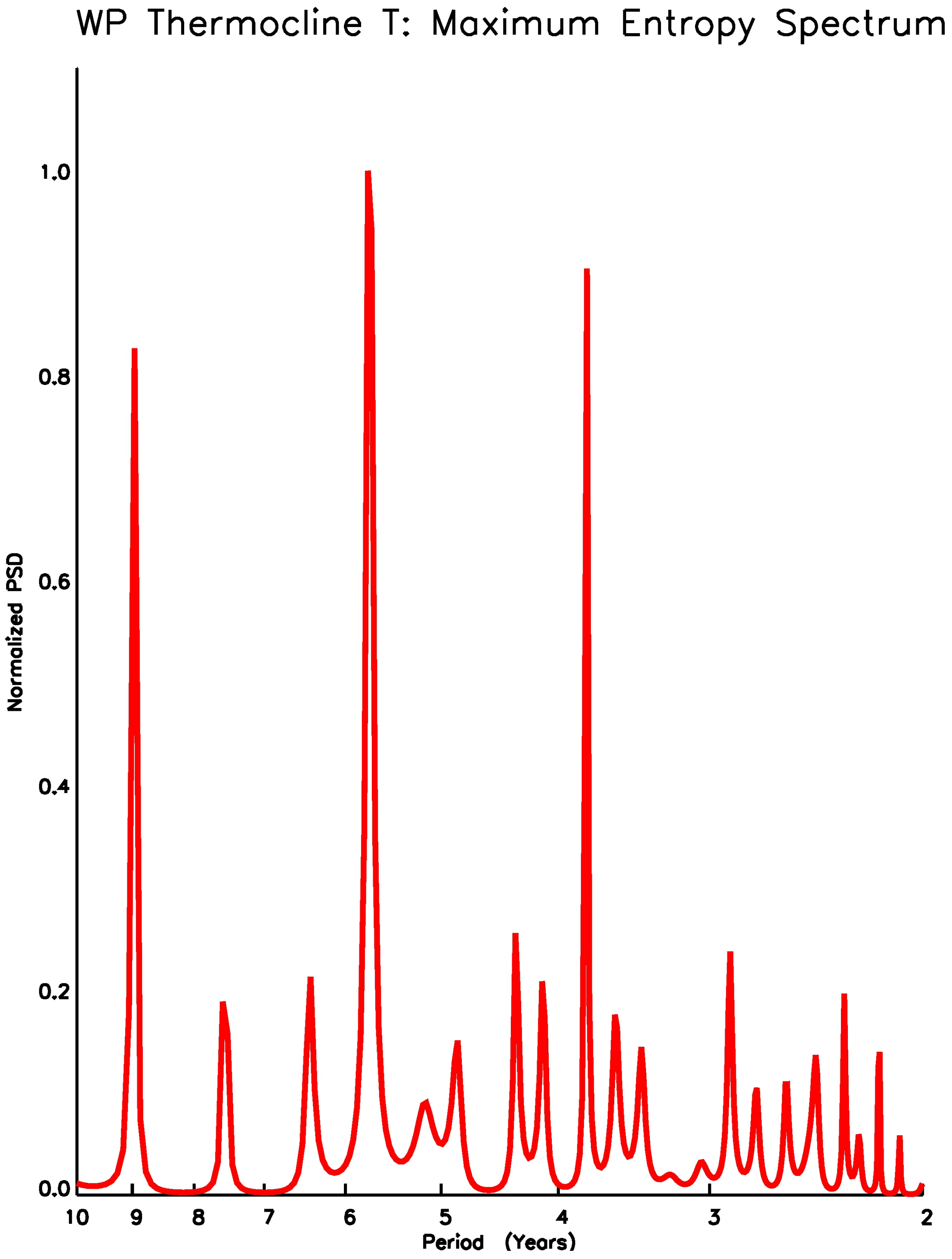

Supplementary Figure 14. Same as Supplementary Figure 13 but for the maximum entropy spectrum of Western Pacific Ocean (5N-5S, 135-140E) subsurface temperature at the thermocline depth (135 m) from the UKMO ocean analysis data.

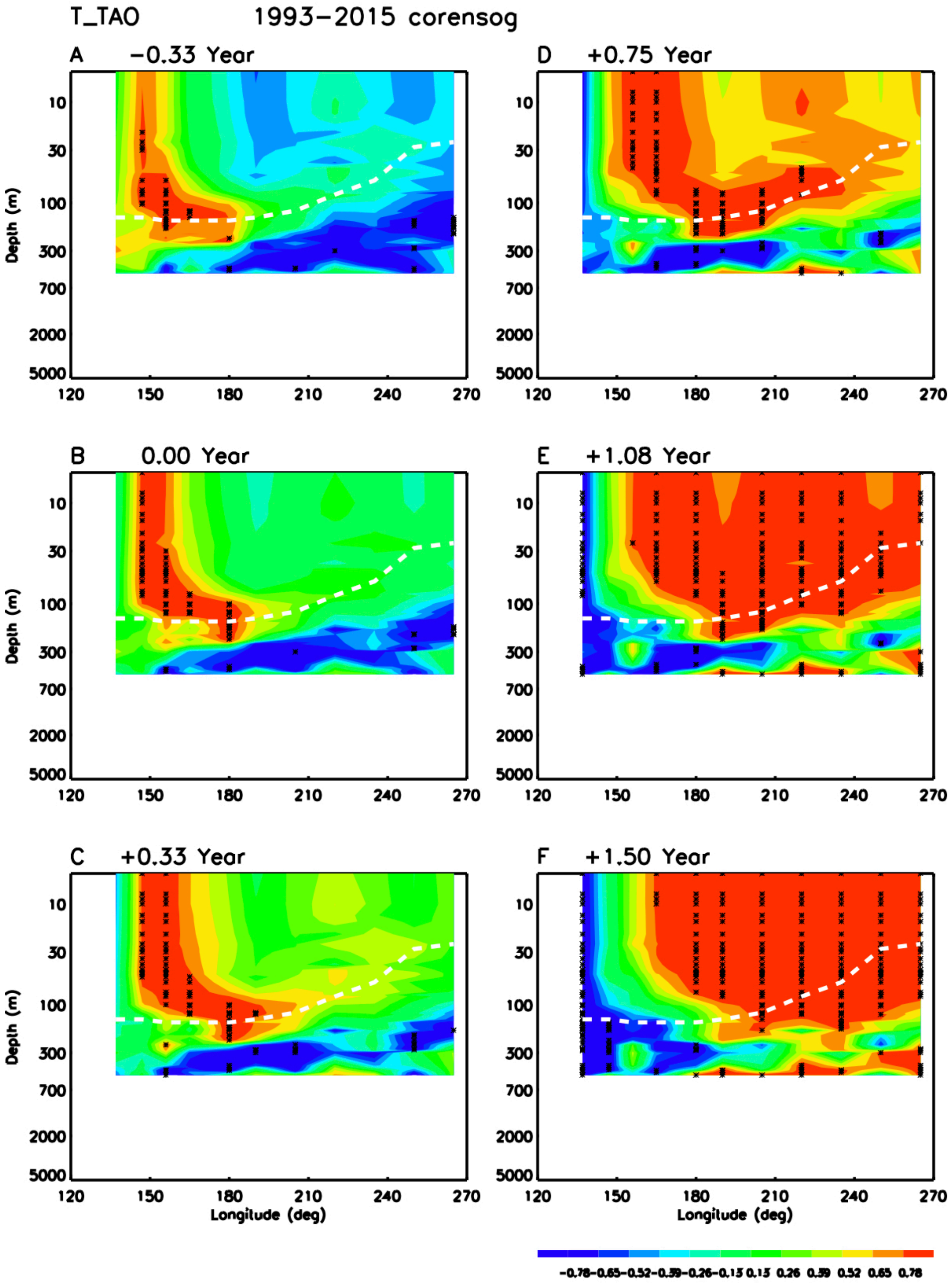

Supplementary Figure 15. Subsurface ocean wave driven by 6-year component of lunar tidal gravitational force. Shadings show lag-correlation of TAO buoy array subsurface temperature along the equator (5N-5S) with lunar tidal force from (A) -0.25 years to (F) +1.5 years. Black stars denote the grids with lag-correlation above 95% confidence level. The white dashed line is the climatological 23.5 ^{o}C line from Supplementary Figure 4a.

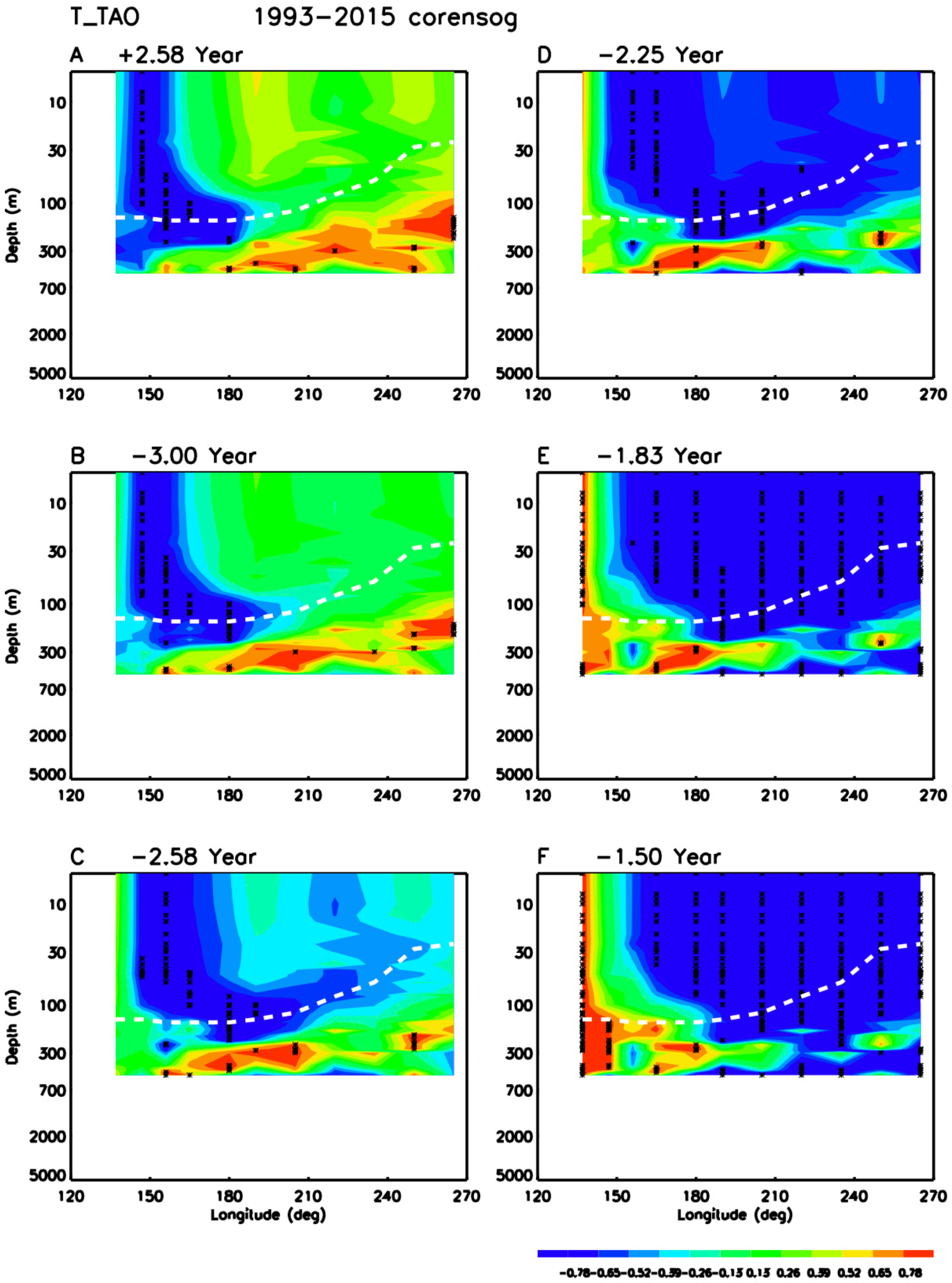

Supplementary Figure 16. Same as Supplementary Figure 15 but for (A) -3.42 years to (F) -1.5 years.
